# (1*S*,4*S*)-2-(2,4-Difluoro­phen­yl)-5-[(4-methyl­phen­yl)sulfon­yl]-2,5-diaza­bicyclo­[2.2.1]hepta­ne

**DOI:** 10.1107/S1600536810054541

**Published:** 2011-01-08

**Authors:** Chunli Wu, Jingyu Zhang, Pan Li, Junxia Zhang, Jizhou Wu

**Affiliations:** a School of Pharmacy, Tongji Medical College, Huazhong University of Science and Technology, Wuhan 430030, People’s Republic of China; b School of Pharmaceutical Sciences, Zhengzhou Univresity, Zhengzhou 450001, People’s Republic of China; c School of Pharmaceutical Sciences, Henan University of TCM, Zhengzhou 450008, People’s Republic of China

## Abstract

In the title mol­ecule, C_18_H_18_F_2_N_2_O_2_S, the two benzene rings, which are oriented in opposite directions with respect to the rigid 2,5-diaza­bicyclo­[2.2.1]heptane core, form a dihedral angle of 17.2 (1)°. Weak inter­molecular C—H⋯O, C—H⋯F and C—H⋯N contacts consolidate the crystal packing.

## Related literature

For details of the synthesis, see: Portoghese *et al.* (1966 ▶); Braish & Fox (1990 ▶); Ulrich *et al.* (1990 ▶). For a recent study of the biological activity of 2,5-diaza­bicyclo­[2.2.1]heptane deriv­atives, see: Li *et al.* (2010 ▶).
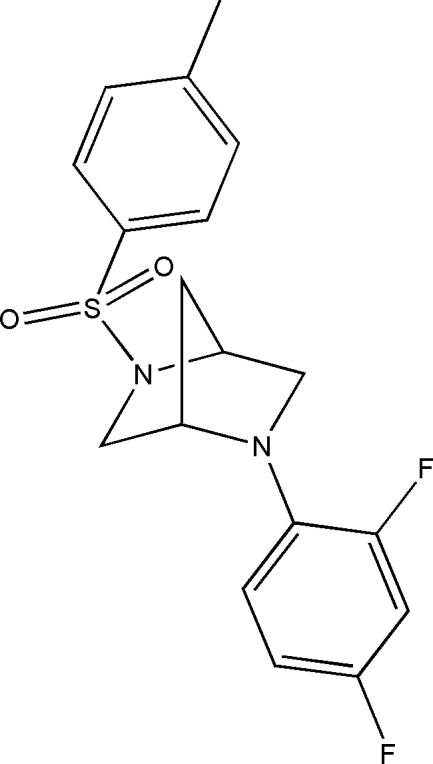


## Experimental

### 

#### Crystal data

C_18_H_18_F_2_N_2_O_2_S
*M* *_r_* = 364.40
Monoclinic, 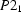

*a* = 9.9615 (11) Å
*b* = 7.6586 (8) Å
*c* = 11.3461 (14) Å
β = 98.979 (1)°
*V* = 855.00 (17) Å^3^
*Z* = 2
Mo *K*α radiation
μ = 0.22 mm^−1^
*T* = 298 K
0.38 × 0.33 × 0.15 mm

#### Data collection

Bruker SMART CCD area-detector diffractometer
Absorption correction: multi-scan (*SADABS*; Sheldrick, 1996 ▶) *T*_min_ = 0.920, *T*_max_ = 0.967
4425 measured reflections
2891 independent reflections
2045 reflections with *I* > 2σ(*I*)
*R*_int_ = 0.028

#### Refinement

*R*[*F* ^2^ > 2σ(*F* ^2^)] = 0.045
*wR*(*F* ^2^) = 0.103
*S* = 1.00
2891 reflections
227 parameters
1 restraint
H-atom parameters constrained
Δρ_max_ = 0.17 e Å^−3^
Δρ_min_ = −0.28 e Å^−3^
Absolute structure: Flack (1983 ▶), 1569 Friedel pairs
Flack parameter: 0.00 (10)

### 

Data collection: *SMART* (Bruker, 2007 ▶); cell refinement: *SAINT* (Bruker, 2007 ▶); data reduction: *SAINT*; program(s) used to solve structure: *SHELXS97* (Sheldrick, 2008 ▶); program(s) used to refine structure: *SHELXL97* (Sheldrick, 2008 ▶); molecular graphics: *SHELXTL* (Sheldrick, 2008 ▶); software used to prepare material for publication: *SHELXL97*.

## Supplementary Material

Crystal structure: contains datablocks I, global. DOI: 10.1107/S1600536810054541/cv5024sup1.cif

Structure factors: contains datablocks I. DOI: 10.1107/S1600536810054541/cv5024Isup2.hkl

Additional supplementary materials:  crystallographic information; 3D view; checkCIF report

## Figures and Tables

**Table 1:** Hydrogen-bond geometry (Å, °)

*D*—H⋯*A*	*D*—H	H⋯*A*	*D*⋯*A*	*D*—H⋯*A*
C3—H3*B*⋯O2^i^	0.97	2.63	3.445 (5)	141
C5—H5*A*⋯O2^i^	0.97	2.70	3.550 (5)	147
C10—H10⋯F1^ii^	0.93	2.63	3.445 (4)	147
C18—H18⋯O1^iii^	0.93	2.43	3.342 (5)	166
C15—H15⋯N2^iv^	0.93	2.66	3.412 (5)	139

Symmetry codes: (i) 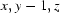; (ii) 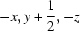; (iii) 
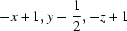
; (iv) 
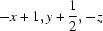
.

**Fig. 1.**
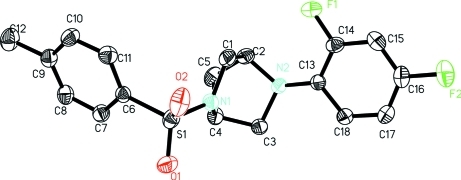
The molecular structure of (I) showing the atomic labels and 30% probability displacement ellipsoids.

**Fig. 2.**
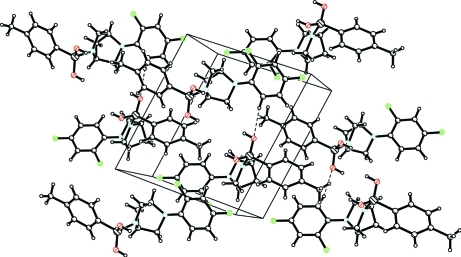
Packing diagram.

## supplementary crystallographic information

### Comment

2,5-Diazabicyclo[2.2.1]heptane derivatives, the synthesis of which is known for a long time (Portoghese *et al.*, 1966; Braish & Fox, 1990), are still under intensive studies. For example, Li *et al.* (2010) used them as novel α7 neuronal nicotinic receptor ligands. Herewith we report the synthesis and crystal structure of the title compound (I) (Fig. 1) prepared in enantiomerically pure form from *trans*-4-hydroxy-*L*-proline (Ulrich *et al.*, 1990).

In (I), the angles C2—C5—C4, C4—N1—C1 and C3—N2—C2 are 92.9 (3), 107.2 (3) and 106.1 (3)°, respectively. The two benzene rings are oriented in opposite directions in reference to the rigid 2,5-diazabicyclo[2.2.1]heptane core, and they form a dihedral angle with the value of 17.2 (1)°. In the crystal structure, weak intramolecular C—H···O, C—H···F and C—H···N hydrogen bonds(Table 1) consolidate the crystal packing.

### Experimental

All reagents and solvents were used as obtained without further purification. (1*S*,4S)-5-(2,4-difluorophenyl)- 2-tosyl-2,5-diazabicyclo[2.2.1]heptane was synthesized from (2*S*,4*R*)-*N*-tosyl-4-tosyloxy-2-tosyloxymethylpyrrolidine as described previously by Ulrich and Fritz, whose started material was *trans*-4-hydroxy-*L*-proline. A solution of 2,4-difluoroaniline(1.5 mL,9.01 mmol) and (2*S*,4*R*)-*N*-tosyl- 4-tosyloxy-2-tosyloxymethylpyrrolidine (0.5 g,0.86 mmol) was refluxed for about 2 h in a 10 ml three-neck bottle until the material was consumed. The resulting mixture was cooled to room tempeature, before ethyl acetate was added. Then the mixture was heated to be able to be stirred and filtered to get the the title compound. m.p.:187–192°C. Crystals suitable for X-ray analysis were grown by slow evaporation from ethyl acetate solution at room temperature for two weeks. The crystals were separated manually. 1H NMR(400 MHz, CDCl3)σ: 7.702–7.681(d,J=8 Hz,2*H*), 7.282–7.263(d,J=7.6 Hz,2*H*), 6.719–6.700(m,J=7.6 Hz,2*H*), 6.448–6.387(m,J=24 Hz,1*H*), 4.463(s,1*H*), 4.339(s,1*H*),3.563–3.539(d,J=9.6 Hz,2*H*), 3.263–3.239(m,J=9.6 Hz,6*H*), 2.415(s,3*H*), 1.845–1.820(d,J=10 Hz,1*H*), 1.374–1.349(d,J=10 Hz,2*H*); 13 C NMR(100.6 MHz,CDCl3)σ: 156.32, 153.94, 143.71, 135.37, 131.70, 129.79, 127.39, 115.76, 110.89, 104.85, 59.87, 59.29, 58.18, 52.31, 36.38, 21.51.

### Refinement

All H atoms were placed geometrically and treated as riding on their parent atoms with C—H are 0.96 Å (methylene) or 0.93 Å (aromatic), 0.82 Å (hydroxyl)and *U*_iso_(H) =1.2*U*_eq_(C).

### Crystal data

**Table:**

C_18_H_18_F_2_N_2_O_2_S	*F*(000) = 380
*M**_r_* = 364.40	*D*_x_ = 1.415 Mg m^−^^3^
Monoclinic, *P*2_1_	Melting point = 460–465 K
Hall symbol: P 2yb	Mo *K*α radiation, λ = 0.71073 Å
*a* = 9.9615 (11) Å	Cell parameters from 1315 reflections
*b* = 7.6586 (8) Å	θ = 3.0–20.7°
*c* = 11.3461 (14) Å	µ = 0.22 mm^−^^1^
β = 98.979 (1)°	*T* = 298 K
*V* = 855.00 (17) Å^3^	Block, colourless
*Z* = 2	0.38 × 0.33 × 0.15 mm

### Data collection

**Table:**

Bruker SMART CCD area-detector diffractometer	2891 independent reflections
Radiation source: fine-focus sealed tube	2045 reflections with *I* > 2σ(*I*)
graphite	*R*_int_ = 0.028
phi and ω scans	θ_max_ = 25.0°, θ_min_ = 2.5°
Absorption correction: multi-scan (*SADABS*; Sheldrick, 1996)	*h* = −10→11
*T*_min_ = 0.920, *T*_max_ = 0.967	*k* = −9→8
4425 measured reflections	*l* = −11→13

### Refinement

**Table:**

Refinement on *F*^2^	Secondary atom site location: difference Fourier map
Least-squares matrix: full	Hydrogen site location: inferred from neighbouring sites
*R*[*F*^2^ > 2σ(*F*^2^)] = 0.045	H-atom parameters constrained
*wR*(*F*^2^) = 0.103	*w* = 1/[σ^2^(*F*_o_^2^) + (0.0457*P*)^2^] where *P* = (*F*_o_^2^ + 2*F*_c_^2^)/3
*S* = 1.00	(Δ/σ)_max_ < 0.001
2891 reflections	Δρ_max_ = 0.17 e Å^−^^3^
227 parameters	Δρ_min_ = −0.28 e Å^−^^3^
1 restraint	Absolute structure: Flack (1983), 1569 Friedel pairs
Primary atom site location: structure-invariant direct methods	Flack parameter: 0.00 (10)

### Special details

**Table:**

Geometry. All e.s.d.'s (except the e.s.d. in the dihedral angle between two l.s. planes) are estimated using the full covariance matrix. The cell e.s.d.'s are taken into account individually in the estimation of e.s.d.'s in distances, angles and torsion angles; correlations between e.s.d.'s in cell parameters are only used when they are defined by crystal symmetry. An approximate (isotropic) treatment of cell e.s.d.'s is used for estimating e.s.d.'s involving l.s. planes.
Refinement. Refinement of *F*^2^ against ALL reflections. The weighted *R*-factor *wR* and goodness of fit *S* are based on *F*^2^, conventional *R*-factors *R* are based on *F*, with *F* set to zero for negative *F*^2^. The threshold expression of *F*^2^ > σ(*F*^2^) is used only for calculating *R*-factors(gt) *etc*. and is not relevant to the choice of reflections for refinement. *R*-factors based on *F*^2^ are statistically about twice as large as those based on *F*, and *R*- factors based on ALL data will be even larger.

### Fractional atomic coordinates and isotropic or equivalent isotropic displacement parameters (Å^2^)

**Table:**

	*x*	*y*	*z*	*U*_iso_*/*U*_eq_	
F1	0.4098 (2)	0.6800 (3)	−0.03940 (17)	0.0765 (8)	
F2	0.8825 (2)	0.6799 (4)	0.0177 (2)	0.1001 (9)	
N1	0.2867 (3)	0.7976 (4)	0.3031 (2)	0.0461 (8)	
N2	0.4040 (3)	0.5378 (4)	0.1917 (2)	0.0433 (7)	
O1	0.2878 (3)	0.9129 (4)	0.5023 (2)	0.0763 (10)	
O2	0.2841 (2)	1.1122 (3)	0.3288 (3)	0.0727 (9)	
S1	0.24048 (8)	0.95563 (13)	0.38078 (9)	0.0536 (3)	
C1	0.2683 (4)	0.8049 (5)	0.1708 (3)	0.0542 (10)	
H1A	0.3421	0.8665	0.1425	0.065*	
H1B	0.1824	0.8583	0.1375	0.065*	
C2	0.2704 (3)	0.6110 (5)	0.1432 (3)	0.0521 (10)	
H2	0.2384	0.5815	0.0595	0.063*	
C3	0.4094 (3)	0.5309 (5)	0.3233 (3)	0.0469 (9)	
H3A	0.4851	0.5981	0.3643	0.056*	
H3B	0.4158	0.4117	0.3525	0.056*	
C4	0.2756 (3)	0.6122 (5)	0.3370 (3)	0.0501 (9)	
H4	0.2454	0.5921	0.4140	0.060*	
C5	0.1836 (4)	0.5390 (6)	0.2296 (3)	0.0607 (10)	
H5A	0.1793	0.4125	0.2293	0.073*	
H5B	0.0928	0.5885	0.2188	0.073*	
C6	0.0621 (3)	0.9624 (5)	0.3636 (3)	0.0417 (8)	
C7	−0.0068 (4)	0.8662 (5)	0.4375 (3)	0.0516 (9)	
H7	0.0408	0.7962	0.4968	0.062*	
C8	−0.1472 (4)	0.8740 (5)	0.4234 (3)	0.0552 (10)	
H8	−0.1931	0.8059	0.4720	0.066*	
C9	−0.2197 (3)	0.9795 (5)	0.3395 (3)	0.0512 (9)	
C10	−0.1492 (4)	1.0745 (5)	0.2650 (3)	0.0556 (10)	
H10	−0.1971	1.1452	0.2063	0.067*	
C11	−0.0098 (4)	1.0664 (5)	0.2761 (3)	0.0528 (10)	
H11	0.0358	1.1306	0.2250	0.063*	
C12	−0.3720 (3)	0.9925 (7)	0.3276 (4)	0.0750 (13)	
H12A	−0.4128	0.9366	0.2550	0.112*	
H12B	−0.3983	1.1132	0.3258	0.112*	
H12C	−0.4021	0.9360	0.3944	0.112*	
C13	0.5230 (3)	0.5761 (4)	0.1463 (3)	0.0415 (9)	
C14	0.5274 (4)	0.6452 (5)	0.0333 (3)	0.0494 (9)	
C15	0.6459 (4)	0.6818 (5)	−0.0094 (3)	0.0578 (11)	
H15	0.6443	0.7309	−0.0845	0.069*	
C16	0.7645 (4)	0.6445 (6)	0.0606 (4)	0.0609 (11)	
C17	0.7683 (4)	0.5735 (6)	0.1699 (4)	0.0633 (12)	
H17	0.8514	0.5473	0.2162	0.076*	
C18	0.6496 (4)	0.5398 (5)	0.2126 (3)	0.0518 (9)	
H18	0.6537	0.4913	0.2882	0.062*	

### Atomic displacement parameters (Å^2^)

**Table:**

	*U*^11^	*U*^22^	*U*^33^	*U*^12^	*U*^13^	*U*^23^
F1	0.0681 (16)	0.109 (2)	0.0488 (13)	0.0138 (14)	−0.0023 (11)	0.0168 (13)
F2	0.0703 (17)	0.134 (3)	0.104 (2)	−0.0124 (16)	0.0408 (14)	0.0063 (17)
N1	0.0493 (18)	0.0448 (19)	0.0458 (18)	0.0052 (16)	0.0121 (14)	0.0034 (15)
N2	0.0394 (16)	0.0426 (17)	0.0466 (17)	0.0031 (13)	0.0027 (13)	−0.0024 (14)
O1	0.0636 (18)	0.099 (3)	0.0592 (17)	0.0166 (16)	−0.0120 (13)	−0.0166 (16)
O2	0.0501 (17)	0.0422 (17)	0.128 (2)	−0.0106 (14)	0.0201 (16)	−0.0099 (17)
S1	0.0412 (5)	0.0511 (6)	0.0669 (7)	−0.0008 (5)	0.0035 (4)	−0.0114 (5)
C1	0.049 (2)	0.056 (3)	0.056 (2)	0.015 (2)	0.0062 (18)	0.012 (2)
C2	0.046 (2)	0.057 (3)	0.049 (2)	−0.0056 (19)	−0.0040 (17)	−0.0054 (19)
C3	0.052 (2)	0.043 (2)	0.046 (2)	0.0041 (17)	0.0080 (17)	0.0080 (16)
C4	0.051 (2)	0.046 (2)	0.056 (2)	0.0032 (18)	0.0165 (18)	0.0146 (18)
C5	0.041 (2)	0.056 (2)	0.083 (3)	−0.0127 (18)	0.004 (2)	0.002 (2)
C6	0.0401 (18)	0.0419 (19)	0.0434 (18)	−0.001 (2)	0.0075 (15)	−0.006 (2)
C7	0.056 (2)	0.046 (2)	0.053 (2)	0.0065 (19)	0.0103 (18)	0.0060 (19)
C8	0.053 (2)	0.051 (2)	0.065 (3)	−0.0021 (19)	0.019 (2)	0.002 (2)
C9	0.0420 (19)	0.053 (3)	0.058 (2)	−0.005 (2)	0.0054 (17)	−0.011 (2)
C10	0.042 (2)	0.063 (3)	0.060 (2)	0.000 (2)	0.0004 (18)	0.006 (2)
C11	0.054 (2)	0.053 (3)	0.051 (2)	−0.0060 (19)	0.0105 (19)	0.0052 (19)
C12	0.046 (2)	0.083 (3)	0.096 (3)	−0.009 (2)	0.010 (2)	−0.014 (3)
C13	0.044 (2)	0.038 (2)	0.042 (2)	0.0035 (16)	0.0062 (16)	−0.0026 (16)
C14	0.055 (2)	0.043 (2)	0.047 (2)	0.0085 (19)	−0.0013 (18)	−0.0001 (19)
C15	0.071 (3)	0.054 (3)	0.051 (2)	−0.001 (2)	0.021 (2)	0.0008 (19)
C16	0.051 (3)	0.063 (3)	0.073 (3)	−0.002 (2)	0.023 (2)	−0.004 (2)
C17	0.041 (2)	0.084 (3)	0.064 (3)	0.003 (2)	0.0040 (19)	0.000 (2)
C18	0.050 (2)	0.059 (2)	0.047 (2)	0.0077 (19)	0.0060 (17)	0.0014 (18)

### Geometric parameters (Å, °)

**Table:**

F1—C14	1.350 (4)	C6—C7	1.378 (4)
F2—C16	1.367 (4)	C6—C11	1.382 (5)
N1—C4	1.480 (4)	C7—C8	1.383 (5)
N1—C1	1.485 (4)	C7—H7	0.9300
N1—S1	1.606 (3)	C8—C9	1.366 (5)
N2—C13	1.395 (4)	C8—H8	0.9300
N2—C2	1.470 (4)	C9—C10	1.387 (5)
N2—C3	1.486 (4)	C9—C12	1.506 (4)
O1—S1	1.424 (3)	C10—C11	1.376 (5)
O2—S1	1.434 (3)	C10—H10	0.9300
S1—C6	1.758 (3)	C11—H11	0.9300
C1—C2	1.518 (5)	C12—H12A	0.9600
C1—H1A	0.9700	C12—H12B	0.9600
C1—H1B	0.9700	C12—H12C	0.9600
C2—C5	1.509 (5)	C13—C18	1.391 (5)
C2—H2	0.9800	C13—C14	1.394 (4)
C3—C4	1.501 (4)	C14—C15	1.372 (5)
C3—H3A	0.9700	C15—C16	1.348 (5)
C3—H3B	0.9700	C15—H15	0.9300
C4—C5	1.514 (5)	C16—C17	1.349 (5)
C4—H4	0.9800	C17—C18	1.371 (5)
C5—H5A	0.9700	C17—H17	0.9300
C5—H5B	0.9700	C18—H18	0.9300
			
C4—N1—C1	107.2 (3)	C7—C6—C11	119.6 (3)
C4—N1—S1	122.8 (2)	C7—C6—S1	120.5 (3)
C1—N1—S1	121.7 (2)	C11—C6—S1	119.9 (3)
C13—N2—C2	123.6 (3)	C6—C7—C8	119.8 (3)
C13—N2—C3	118.6 (3)	C6—C7—H7	120.1
C2—N2—C3	106.1 (3)	C8—C7—H7	120.1
O1—S1—O2	120.99 (18)	C9—C8—C7	121.4 (3)
O1—S1—N1	106.15 (17)	C9—C8—H8	119.3
O2—S1—N1	105.85 (14)	C7—C8—H8	119.3
O1—S1—C6	106.89 (15)	C8—C9—C10	118.2 (3)
O2—S1—C6	107.21 (17)	C8—C9—C12	121.2 (4)
N1—S1—C6	109.44 (16)	C10—C9—C12	120.6 (4)
N1—C1—C2	99.7 (3)	C11—C10—C9	121.4 (3)
N1—C1—H1A	111.8	C11—C10—H10	119.3
C2—C1—H1A	111.8	C9—C10—H10	119.3
N1—C1—H1B	111.8	C10—C11—C6	119.6 (3)
C2—C1—H1B	111.8	C10—C11—H11	120.2
H1A—C1—H1B	109.6	C6—C11—H11	120.2
N2—C2—C5	101.2 (3)	C9—C12—H12A	109.5
N2—C2—C1	109.7 (3)	C9—C12—H12B	109.5
C5—C2—C1	101.3 (3)	H12A—C12—H12B	109.5
N2—C2—H2	114.4	C9—C12—H12C	109.5
C5—C2—H2	114.4	H12A—C12—H12C	109.5
C1—C2—H2	114.4	H12B—C12—H12C	109.5
N2—C3—C4	101.3 (2)	C18—C13—C14	114.7 (3)
N2—C3—H3A	111.5	C18—C13—N2	120.6 (3)
C4—C3—H3A	111.5	C14—C13—N2	124.7 (3)
N2—C3—H3B	111.5	F1—C14—C15	117.1 (3)
C4—C3—H3B	111.5	F1—C14—C13	119.2 (3)
H3A—C3—H3B	109.3	C15—C14—C13	123.6 (3)
N1—C4—C3	105.5 (3)	C16—C15—C14	118.2 (3)
N1—C4—C5	101.9 (3)	C16—C15—H15	120.9
C3—C4—C5	101.5 (3)	C14—C15—H15	120.9
N1—C4—H4	115.4	C15—C16—C17	121.6 (4)
C3—C4—H4	115.4	C15—C16—F2	118.1 (4)
C5—C4—H4	115.4	C17—C16—F2	120.3 (4)
C2—C5—C4	92.9 (3)	C16—C17—C18	119.9 (4)
C2—C5—H5A	113.1	C16—C17—H17	120.0
C4—C5—H5A	113.1	C18—C17—H17	120.0
C2—C5—H5B	113.1	C17—C18—C13	122.0 (3)
C4—C5—H5B	113.1	C17—C18—H18	119.0
H5A—C5—H5B	110.5	C13—C18—H18	119.0
			
C4—N1—S1—O1	42.5 (3)	O2—S1—C6—C11	21.9 (3)
C1—N1—S1—O1	−173.3 (3)	N1—S1—C6—C11	−92.5 (3)
C4—N1—S1—O2	172.2 (3)	C11—C6—C7—C8	0.2 (5)
C1—N1—S1—O2	−43.5 (3)	S1—C6—C7—C8	179.5 (3)
C4—N1—S1—C6	−72.6 (3)	C6—C7—C8—C9	−2.0 (5)
C1—N1—S1—C6	71.7 (3)	C7—C8—C9—C10	2.5 (6)
C4—N1—C1—C2	−8.7 (3)	C7—C8—C9—C12	−177.7 (4)
S1—N1—C1—C2	−157.7 (2)	C8—C9—C10—C11	−1.3 (5)
C13—N2—C2—C5	−175.9 (3)	C12—C9—C10—C11	178.9 (3)
C3—N2—C2—C5	−33.6 (3)	C9—C10—C11—C6	−0.4 (5)
C13—N2—C2—C1	−69.4 (4)	C7—C6—C11—C10	0.9 (5)
C3—N2—C2—C1	72.9 (4)	S1—C6—C11—C10	−178.4 (3)
N1—C1—C2—N2	−63.7 (3)	C2—N2—C13—C18	163.7 (3)
N1—C1—C2—C5	42.7 (3)	C3—N2—C13—C18	25.7 (5)
C13—N2—C3—C4	141.8 (3)	C2—N2—C13—C14	−18.8 (5)
C2—N2—C3—C4	−2.7 (3)	C3—N2—C13—C14	−156.8 (3)
C1—N1—C4—C3	77.7 (3)	C18—C13—C14—F1	178.0 (3)
S1—N1—C4—C3	−133.7 (3)	N2—C13—C14—F1	0.4 (5)
C1—N1—C4—C5	−28.0 (3)	C18—C13—C14—C15	−2.4 (5)
S1—N1—C4—C5	120.6 (3)	N2—C13—C14—C15	180.0 (3)
N2—C3—C4—N1	−67.9 (3)	F1—C14—C15—C16	−178.5 (3)
N2—C3—C4—C5	38.0 (3)	C13—C14—C15—C16	1.8 (6)
N2—C2—C5—C4	54.4 (3)	C14—C15—C16—C17	−0.1 (6)
C1—C2—C5—C4	−58.5 (3)	C14—C15—C16—F2	179.6 (4)
N1—C4—C5—C2	51.9 (3)	C15—C16—C17—C18	−0.9 (6)
C3—C4—C5—C2	−56.9 (3)	F2—C16—C17—C18	179.4 (4)
O1—S1—C6—C7	−26.3 (3)	C16—C17—C18—C13	0.2 (6)
O2—S1—C6—C7	−157.4 (3)	C14—C13—C18—C17	1.3 (5)
N1—S1—C6—C7	88.2 (3)	N2—C13—C18—C17	179.0 (3)
O1—S1—C6—C11	153.0 (3)		

### Hydrogen-bond geometry (Å, °)

**Table:**

*D*—H···*A*	*D*—H	H···*A*	*D*···*A*	*D*—H···*A*
C3—H3B···O2^i^	0.97	2.63	3.445 (5)	141
C5—H5A···O2^i^	0.97	2.70	3.550 (5)	147
C10—H10···F1^ii^	0.93	2.63	3.445 (4)	147
C18—H18···O1^iii^	0.93	2.43	3.342 (5)	166
C15—H15···N2^iv^	0.93	2.66	3.412 (5)	139

Symmetry codes: (i) *x*, *y*−1, *z*; (ii) −*x*, *y*+1/2, −*z*; (iii) −*x*+1, *y*−1/2, −*z*+1; (iv) −*x*+1, *y*+1/2, −*z*.

## References

[bb1] Braish, T. F. & Fox, D. E. (1990). *J. Org. Chem.* **55**, 1684–1687.

[bb2] Bruker (2007). *SMART* and *SAINT* Bruker AXS Inc., Madison, Wisconsin, USA.

[bb3] Flack, H. D. (1983). *Acta Cryst.* A**39**, 876–881.

[bb4] Li, T., Bunnelle, W. H., Ryther, K. B., Anderson, D. J., Malysz, J., Helfrich, R., Granlien, J. H., Håkerud, M., Peters, D., Schrimpf, M. R., Gopalakrishnan, M. & Ji, J. G. (2010). *Bioorg. Med. Chem. Lett.* **20**, 3636–3639. 10.1016/j.bmcl.2010.04.105 20472430

[bb5] Portoghese, P. S., Larson, D. L. & Takemori, A. E. (1966). *Eur. J. Pharmacol.* **4**, 445–451. 10.1016/0014-2999(81)90355-1 6263637

[bb6] Sheldrick, G. M. (1996). *SADABS* University of Göttingen, Germany.

[bb7] Sheldrick, G. M. (2008). *Acta Cryst.* A**64**, 112–122. 10.1107/S0108767307043930 18156677

[bb8] Ulrich, J., Fritz, S., Suhaib, M. S., Bernhard, K. & Kaberi, B. (1990). *Synthesis*, **11**, 925–930.

